# A distinctive gene expression fingerprint in mentally retarded male patients reflects disease-causing defects in the histone demethylase KDM5C

**DOI:** 10.1186/1755-8417-3-2

**Published:** 2010-02-02

**Authors:** Lars R Jensen, Heinz Bartenschlager, Sinitdhorn Rujirabanjerd, Andreas Tzschach, Astrid Nümann, Andreas R Janecke, Ralf Spörle, Sigmar Stricker, Martine Raynaud, John Nelson, Anna Hackett, Jean-Pierre Fryns, Jamel Chelly, Arjan PM de Brouwer, Ben Hamel, Jozef Gecz, Hans-Hilger Ropers, Andreas W Kuss

**Affiliations:** 1 Department of Human Molecular Genetics, Max Planck Institute for Molecular Genetics, Berlin, Germany; 2 Department of Animal Breeding and Biotechnology, University of Hohenheim, Stuttgart, Germany; 3 Department of Molecular Pathology, SA Pathology and Women's and Children's Hospital, Adelaide, South Australia, Australia; 4 Genetische Poliklinik, Klinikum der Universität Heidelberg, Heidelberg, Germany; 5 Division of Clinical Genetics, Innsbruck Medical University, Innsbruck, Austria; 6 Department of Developmental Genetics, Max Planck Institute for Molecular Genetics, Berlin, Germany; 7 Development and Disease Group, Max Planck Institute for Molecular Genetics, Berlin, Germany; 8 CHRU de Tours, Service de Génétique, 37000 Tours; INSERM U930, 37000 Tours, France; 9 Genetic Services of Western Australia, King Edward Memorial Hospital for Women, Perth, Western Australia, Australia; 10 The GOLD Service, Hunter Genetics, Waratah, New South Wales, Australia; 11 Centre for Human Genetics, University Hospital Leuven, Leuven, Belgium; 12 Institut Cochin de Génétique Moléculaire, Centre National de la Recherche Scientifique (CNRS), Paris, France; 13 Department of Human Genetics, Radboud University Nijmegen Medical Center, Nijmegen, The Netherlands; 14 Department of Paediatrics, University of Adelaide, Adelaide, Australia

## Abstract

**Background:**

Mental retardation is a genetically heterogeneous disorder, as more than 90 genes for this disorder has been found on the X chromosome alone. In addition the majority of patients are non-syndromic in that they do not present with clinically recognisable features. This makes it difficult to determine the molecular cause of this disorder on the basis of the phenotype alone. Mutations in *KDM5C *(previously named *SMCX *or *JARID1C*), a gene that encodes a transcriptional regulator with histone demethylase activity specific for dimethylated and trimethylated H3K4, are a comparatively frequent cause of non-syndromic X-linked mental retardation (NS-XLMR). Specific transcriptional targets of KDM5C, however, are still unknown and the effects of KDM5C deficiency on gene expression have not yet been investigated.

**Results:**

By whole-mount *in situ *hybridisation we showed that the mouse homologue of *KDM5C *is expressed in multiple tissues during mouse development.

We present the results of gene expression profiling performed on lymphoblastoid cell lines as well as blood from patients with mutations in *KDM5C*. Using whole genome expression arrays and quantitative reverse transcriptase polymerase chain reaction (QRT-PCR) experiments, we identified several genes, including *CMKOR1*, *KDM5B *and *KIAA0469 *that were consistently deregulated in both tissues.

**Conclusions:**

Our findings shed light on the pathological mechanisms underlying mental retardation and have implications for future diagnostics of this heterogeneous disorder.

## Background

Moderate to severe mental retardation (MR) affects 0.5% of the population in developed countries, and in about 10% of these patients the causative mutations are thought to be located on the X chromosome [1]. Based on the phenotype, X-linked mental retardation (XLMR) is often subdivided into syndromic forms characterised by additional clinical symptoms and non-syndromic forms where MR is the only clinically consistent feature (NS-XLMR). Non-syndromic forms seem to be more prevalent and they show a high degree of genetic heterogeneity; defects in >30 genes have been reported in patients with NS-XLMR.

In the absence of specific clinical phenotypes, these individual disease entities can only be distinguished by molecular studies. However, sequencing a large number of candidate genes to identify the underlying molecular defects is too costly and time consuming for routine diagnostics, at least with the currently employed methodology [2]. Moreover, very often this approach will not discriminate between disease-causing mutations and functionally neutral changes. This is a crucial issue because in a majority of cases, the resequencing of candidate genes leads to the detection of missense changes with unclear functional relevance, as shown by a recent study of >700 Vega-annotated X-chromosomal genes in a cohort of 200 NS-XLMR patients [3]. Tests, however, that are suitable for discerning disease-causing mutations are only available for a small minority of the known NS-XLMR genes so far. However, if such tests do not exist, gene expression profiling is an alternative, provided the candidate gene is expressed in accessible cells or tissues. Such an approach has been pioneered by Cheung *et al*. [4] who found recognisable expression changes in carriers of mutations in the Nijmegen Breakage Syndrome gene, even though this gene is not directly involved in transcription. In contrast *KDM5C *(also known as *JARID1C *and *SMCX*), one of the more frequently mutated genes in XLMR [5-8], encodes a transcription factor that possesses several DNA binding motifs and shows histone demethylation activity specific for dimethylated and trimethylated lysine 4 of histone H3 [9-11]. Overexpression studies using a range of *KDM5C *cDNA constructs with different previously reported mutations revealed reduced histone demethylase activity [10,11] (Additional file 1). Reduction of demethylase activity should have transcriptional consequences [11], but specific transcriptional targets and effects of KDM5C deficiency are not yet known. We therefore performed whole genome expression profiling and quantitative reverse transcriptase polymerase chain reaction (QRT-PCR) experiments using RNA from lymphoblastoid cell lines or blood from patients carrying loss of function mutations or potentially damaging missense changes in KDM5C. We have identified several genes that are deregulated in the absence of KDM5C function shedding more light on the function of KDM5C in gene regulation. Moreover, we found that these patients have specific gene expression signatures, which could be used to assess the pathogenic nature of DNA changes in *KDM5C*.

## Results

### Whole mount *in situ *hybridisation in mouse embryos

Northern blot analysis showed expression of *KDM5C *in all human tissues investigated to date, including the brain, heart, skeletal muscle, liver, pancreas and lung [5]. To investigate expression in a wider range of mammalian tissues we performed whole mount *in situ *hybridisation on mouse embryos using a *KDM5C *specific probe (Additional file 2a-d), which revealed widespread expression at all investigated developmental stages (Theiler stages TS12, TS15, TS17 and TS18). At TS21 (embryonic day 12.5) we performed sagittal section hybridisations and observed higher expression levels in several neuronal tissues including the spinal ganglia, the neural ectoderm, differentiated neurons of the spinal cord and the telencephalon as well as in the liver and the heart (Additional file 2e, f).

### Gene expression analysis

KDM5C is a transcription factor. As such it is likely that deleterious changes in *KDM5C *give rise to specific transcriptional effects, which might help to explain why they are disease causing. To search for deregulated transcripts we performed genome-wide expression analysis using Epstein Barr virus (EBV) transformed patient B lymphocytes (LCL). The patient cell line used for this initial expression-profiling step was obtained from a patient carrying KDM5C Trp1288Ter mutation. We have previously shown that this mutation leads to reduced *KDM5C *expression [5]. Subsequently we individually compared the expression profile of this LCL with each one of three different controls and extracted highly expressed genes (detection threshold >0.99), which also showed strong differential expression (differential expression score of >50) and excluded genes that showed less than twofold difference in the expression level between patient and control cell lines. This led to the identification of 21 genes that showed consistent differential expression (Table 1). In addition, we found that three other genes also showed strong differential expression between the patient and two of the three controls from the analysis: *KDM5B *(also known as *JARID1B*), *RPS6KA3 *and *MKNK2*.

**Table 1:** 21 genes selected for expression studies by QRT-PCR.

DS>100	DS>50
DT>0.99	DT = 1
*Hs.136376*	*OXTR*
*MGC20983*	*SLAMF6*
*APM2*	*MSL3L1*
*MYC*	*KIAA0469*
*CETP*	*SLC1A4*
*LOC148918*	
*CMKOR1*	
*MSL3L1*	
*SQSTM1*	
*CD55*	
*EMILIN2*	
*NEUROG2*	
*HSPA1B*	
*CABP1*	
*OAS3*	
*TNFSF4*	

DS: Differential score, DT: detection threshold

Subsequently we performed QRT-PCR for all 24 genes using RNA from the original patient lymphoblastoid cell line plus 3 control lymphoblastoid cell lines and confirmed our observations from the array analysis for 22 out of 24 genes. Only two genes (Hs. 136376 and Neurog2) did not yield the minimum amount of PCR product required for quantitative measurements, due to extremely low expression levels.

To further validate this set of 22 genes we expanded the QRT-PCR analysis to 12 lymphoblastoid cell lines from unrelated patients with KDM5C mutations and 5 controls. Quantified Northern blot analyses for the four differentially expressed genes (*CMKOR1*, *MKNK2*, *MYC *and *KDM5B*) confirmed the original findings (Additional file 3). Of the 12 patients, 4 carried premature termination codon (PTC) mutations and 8 had missense mutations [5,7]. The protein truncating mutations are most likely to cause a loss of KDM5C function. The pathogenic relevance of missense mutations, however, is more difficult to assess. To perform the analysis we grouped our QRT-PCR data according to mutation type, analysed the results separately and then searched for commonly deregulated genes. Of the 22 genes identified by array analysis and confirmed by QRT-PCR, 7 genes were significantly deregulated (Student t test: *P *< 0.05) in both groups (Table 2) while 5 genes showed expression differences that depended on the nature of the *KDM5C *mutation (missense or truncating).

**Table 2:** Differentially expressed genes in lymphoblastoid cell lines from MR patients with changes in *KDM5C*.

			Missense change		Truncating mutation	
				
Symbol	Protein	RefSeq ID	Ratio^a^	Significance^b^	Ratio^a^	Significance^c^
CETP^d^	cholesteryl ester transfer protein, plasma	NM_000078.1	4.34	NS	8.57	*
CD55^e^	decay accelerating factor for complement	NM_000574.2	2.03	*	1.88	NS
CMKOR1^f^	chemokine orphan receptor 1	NM_020311.1	6.85	**	6.29	*
EMILIN2^f^	elastin microfibril interfacer 2	NM_032048.1	2.34	***	2.49	***
HSPA1B^e^	heat shock 70 kDa protein 1B	NM_005346.3	4.00	*	2.66	NS
KDM5B^e^	lysine (K)-specific demethylase 5B	NM_006618.2	3.18	***	1.94	NS
KIAA0469^d^	KIAA0469	XM_375685.1	1.52	NS	1.74	*
MGC20983^f^	hypothetical protein LOC115948	NM_145045.3	2.72	*	2.94	*
MKNK2^f^	MAP kinase-interacting serine/threonine kinase 2	NM_017572.2	2.07	*	2.40	**
MYC^f^	myc proto-oncogene protein	NM_002467.2	2.54	**	2.89	**
SLAMF6^f^	activating NK receptor precursor	NM_052931.3	2.71	**	2.41	*
TNFSF4^f^	tumor necrosis factor (ligand) superfamily	NM_003326.2	0.20	**	0.12	**

NS: Not significant; *: P < 0.05; **: P < 0.01; ***: P < 0.001

^a^) Ratio was calculated as the mean expression divided by mean control expression

^b^) Level of significance was calculated by students' t-test using expression data from carriers of missense changes (n = 8)

^c^) Level of significance was calculated by students' t-test using expression data from carriers of truncating mutations (n = 4)

^d^) Genes significantly deregulated in truncating mutation carriers

^e^) Genes significantly deregulated in missense change carriers

^f^)7 genes that are significantly deregulated for both mutation types are marked in bold

### mRNA expression profiling as a diagnostic tool

We were interested to investigate whether there was a gene expression signature typical and discriminatory for the disease-causing changes in KDM5C.

**Figure 1:**
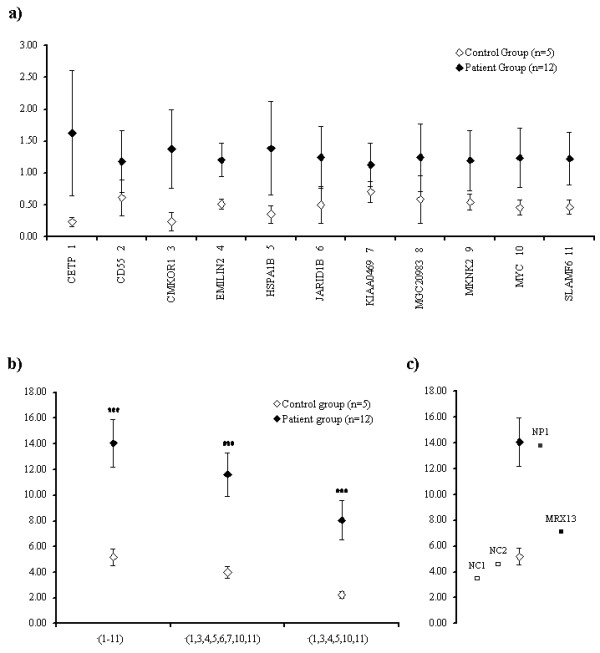
**Weighted accumulative permutation analysis for differentiation between groups (WAPDG) results**. **(a) **Means of the 1,000-fold permutated expression values per gene in patients and controls. **(b) **Results of summarised analysis including different subsets of genes. **(c) **Results of WAPDG analysis of two novel patients (NP1 and MRX13) and two novel controls (NC1 and NC2). The error bars represent the SD.

To test the hypothesis, we developed and performed the method of statistical weighted accumulative permutation analysis for differentiation between groups (WAPDG). The results of this analysis showed that the observations from some individual genes were not significantly different between patients and controls (Figure 1a). However, the combined expression data from all 11 upregulated genes (see Table 2) were significantly different and allowed us to distinguish patients from controls with a high degree of confidence (*P *< 0.001) (Figure 1b). These data also suggests that even smaller subsets of the selected genes (for example, *CETP*, *CMKOR1*, *EMILIN2*, *HSPA1B*, *MYC *and *SLAMF6*) are sufficient to differentiate between patients and controls at a significance level of *P *< 0.001 (Figure 1b).

To test the discriminatory power of our gene expression signature and to evaluate whether the expression levels of these genes are sensitive to cell culture handling, we applied our approach in a blind study, using lymphoblastoid cell lines from two unrelated patients with recently ascertained novel KDM5C mutations [12] and two additional controls, all of which had been grown in a different laboratory. The WAPDG method allowed us to correctly assign the two controls and to unequivocally determine patient status for one sample (NP1). The remaining patient's (MRX13) expression signatures could not be assigned to either control or patient groups (Figure 1c).

Since the selection of genes used in this study had so far been based on the expression profiling results from a single patient and three controls, we also wanted to determine whether the selected genes were among the most highly differentially expressed in additional patients and controls. We therefore compared our results with genome-wide expression analyses of all 12 patients and 5 controls. The results of these experiments validated our original selection of marker genes, as they showed that with the exception of *MKNK2 *all previously selected and analysed genes were among the top 1% of significantly deregulated genes in the expanded group of patients.

### Differential mRNA expression in whole blood from MR patients and obligate carriers

**Figure 2:**
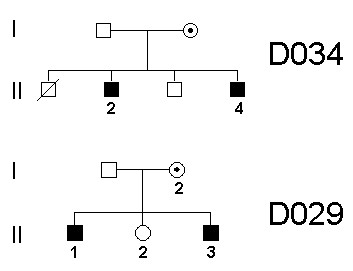
**mRNA expression in whole blood from two mental retardation (MR) families**. The pedigrees of two MR families for which whole blood was obtained are shown. Affected members of family D034 carry the frameshift mutation R68fsX7 and affected members of family D029 carry a Glu698Lys missense mutation. Numbers indicate the individuals from whom whole blood was obtained for RNA extraction.

To further validate the gene expression signature obtained from lymphoblastoid cell lines we performed QRT-PCR on mRNA isolated from whole blood from affected members of two families. These families (D034 and D029) carry a protein truncating mutation (R68fsX7) and a missense change (Glu698Lys), respectively [5] (Figure 2). As controls we used RNA extracted from the blood of five healthy male individuals.

In all, 10 out of 12 genes that were differentially expressed in LCLs were also found to be expressed in blood and 3 of these (*CMKOR1*, *KDM5B *and *KIAA0469*) were significantly upregulated (*P *< 0.05, Student t test) in all affected individuals from both families (Table 3).

**Table 3:** Differential mRNA expression in whole blood from MR patients.

			Missense change		Truncating mutation	
				
Symbol		Sequence ID	Ratio^a^	Significance^b^	Ratio^a^	Significance^c^
CETP^d^	cholesteryl ester transfer protein, plasma	NM_000078.1		ND		ND
CD55^e^	decay accelerating factor for complement	NM_000574.2	7.50	**	1.00	NS
CMKOR1^f^	chemokine orphan receptor 1	NM_020311.1	6.30	***	3.40	***
EMILIN2	elastin microfibril interfacer 2	NM_032048.1	1.18	NS	0.82	NS
HSPA1B^e^	heat shock 70 kDa protein 1B	NM_005346.3	2.71	*	1.34	NS
KDM5B^f^	lysine (K)-specific demethylase 5B	NM_006618.2	3.74	***	1.58	*
KIAA0469^f^	KIAA0469	XM_375685.1	3.22	***	2.04	**
MGC20983^d^	hypothetical protein LOC115948	NM_145045.3		ND		ND
MKNK2^e^	MAP kinase-interacting serine/threonine kinase 2	NM_017572.2	3.65	***	1.27	NS
MYC	myc proto-oncogene protein	NM_002467.2	1.20	NS	0.75	NS
SLAMF6	activating NK receptor precursor	NM_052931.3	1.09	NS	1.08	NS
TNFSF4^e^	tumor necrosis factor (ligand) superfamily	NM_003326.2	1.66	*	0.99	NS

ND: Not determined; NS: Not significant; *: P < 0.05; **: P < 0.01; ***: P < 0.001

^a^) Ratio was calculated as the mean expression from affected in the family divided by mean control expression

^b^) Level of significance was calculated by students' t-test using expression data from patients with missense a change (n = 2) and controls (n = 5)

^c^) Level of significance was calculated by students' t-test using expression data from patients premature termination (n = 2) and controls (n = 5)

^d^)Genes with too low expression for reliable QRT-PCR evaluation

^e^) Genes significantly deregulated in missense change carriers

^f^) 3 genes that are significantly deregulated in affected from both families are marked in bold

## Discussion

In this study we performed genome-wide gene expression analyses in order to identify genes that are significantly upregulated or downregulated in LCLs and blood from patients with mutations in the transcription factor KDM5C. This led to the identification of 12 genes that are significantly deregulated in patient LCLs. Seven genes were consistently deregulated in LCLs from patients with missense and nonsense mutations and it is tempting to speculate that deregulation of at least some of these putative KDM5C targets is involved in the aetiology of MR in the patients.

Most of the differentially expressed genes observed in the genome-wide analysis show upregulation in patient LCLs. This is remarkable, as the proportion of genes with elevated expression exceeds by far what had to be expected due to the normal detection bias towards upregulated genes in such experiments, and it suggests that KDM5C acts primarily as a transcriptional repressor, which is in fact in line with recent studies reporting repression of Smad3 activity by KDM5C [13].

The seven missense changes investigated in this study have, for all but one, been shown to result in reduced demethylase activity *in vitro *or in altered cellular localisation [10,11]. The remaining missense change (Arg750Trp) is located in the C5HC2 zinc finger domain, immediately adjacent to a missense change (Tyr751Cys) with a known effect on demethylase activity [10] and can thus be assumed to cause a similar impairment of KDM5C function. All these missense changes are therefore very likely causative mutations. The expression pattern of the 12 selected genes in these missense changes also correlates better with that of the cell lines with truncating mutations than with that of the controls. For several genes (*CD55*, *CMKOR1*, *HSPA1B*, *KDM5B*, and *SLAMF6*) missense changes seem to have a stronger effect on the expression level changes as compared to truncating mutations. This might be due to negative gain of function effects where mutated KDM5C could, for example, obstruct other demethylase enzymes in compensating for the loss of physiological KDM5C function. Therefore, expression profiling may be a valuable tool for testing the relevance of missense changes in *KDM5C *as patients with mutations in *KDM5C *do not necessarily present with other consistent, clinically identifiable features. From a practical and diagnostic point of view this approach may be more feasible than using currently available histone demethylase assay, which involves cloning and mass spectrometry.

It appears that the expression profile we identified correlates with the severity of the molecular and/or clinical patient phenotype. This became evident in two cases where the expression profile did not allow unequivocal assignment of the affected status, namely the patient from family MRX13 (Figure 1c) and the patient from P081 with a Phe642Leu change. In these two cases our gene expression results reflect the relatively mild impairment of histone 3 lysine 4 demethylase activity [10] observed in the patient carrying the Phe642Leu change and the intermediate clinical phenotype observed in MRX13 [12].

LCLs were previously shown to provide a useful model for the investigation of the expression of genes that are relevant in human cognition [14]. However, as whole blood is more easily accessible in a diagnostic setting we compared the expression patterns obtained from LCLs with those observed in blood-derived RNA preparations from patients of two families with different *KDM5C *mutation types. We thus found that *KDM5B*, *CMKOR1 *and *KIAA0469 *were consistently upregulated in LCLs as well as whole blood from all affected individuals. *KDM5B *is particularly interesting because it encodes a histone 3 lysine 4 demethylase that is highly similar to *KDM5C *[9,15]. It is therefore possible that KDM5B can partially compensate for KDM5C deficiency in most tissues, but not in a manner that is sufficient to ensure normal brain development and function. This could explain why the patients do not show any other clinical features apart from MR.

The second gene consistently deregulated in blood and LCLs from patients with *KDM5C *mutations is the chemokine receptor-encodinggene *CMKOR1*/*CXCR7*. Upregulation of this gene has also been found in lymphoblastoid cell lines from autistic patients with FMR1 mutations or 15q11-q13 duplications [14]. Interestingly, several other genes that we found to be deregulated in KDM5C deficient cell lines showed similarly, albeit less significantly altered expression in a previous study [14]. These genes include *GPR155 *(G protein coupled receptor 155), *PITPNC1 *(phosphatidylinositol transfer protein, cytoplasmic, 1), *LCP2 *(lymphocyte cytosolic protein 2) and *HIG2 *(hypoxia-inducible protein 2). Together with the recent finding of a *KDM5C *missense change in a patient with autism spectrum disorder [8], our findings suggest that autism and non-syndromic MR may overlap at the phenotypical as well as the molecular level.

*KIAA0469*/*KLHL21 *(Kelch-like 21) encodes a brain expressed but otherwise uncharacterised protein. Other members of the KLHL family of proteins are involved in protein degradation [16] and in neurite outgrowth [17]. Since proper functioning of the central nervous system depends on the unperturbed formation of interneuronal connections, our observation that *KIAA0469*/*KLHL21 *is deregulated in MR patients with KDM5C mutations strongly suggests that its function is similar to that of the other members of the KLHL family. This result provides a starting point for future research into the function of *KIAA0469*/*KLHL21 *and also suggests that KDM5C deficiency may involve neurite outgrowth.

## Conclusions

We have shown that *KDM5C *is ubiquitously expressed during the early stages of mammalian ontogenesis and provide data describing the transcriptional effects of KDM5C deficiency in both lymphoblastoid cell lines and whole blood from mutation carriers and healthy individuals. Our study points out the potential of expression profiling for diagnostic purposes in XLMR, by providing a method for testing the pathogenic relevance of missense mutations in this gene. Finally our results shed light on the function of KDM5C as a transcriptional repressor and open up new avenues of research for elucidating the causes of mental retardation in patients with KDM5C deficiency.

## Methods

### Patients

Lymphoblastoid cell lines from the index patients of 12 different MR families were used in this study. Blood was obtained from members of the MR families D029 and D034 carrying a Glu698Lys and an R68fsX7 mutation, respectively. All patients had NS-XLMR without consistent additional features and have been described elsewhere [5,7]. For all affected families with patients carrying missense changes, cosegregation with the disorder had been shown except for family P081, where no other family members were available [5,7].

The probands or their parents gave their written informed consent to these investigations. The study was approved by the institutional review board of the University Hospital Berlin (Charité), Berlin, Germany.

### Mouse *in situ *hybridisation

A PCR fragment of 825 base pairs (bp), corresponding to position 4,416 to 5,240 of the mouse NM_013668.2 reference sequence was amplified from cDNA using the primers: Smcx_ISH_1f 5'-GGTACGAAGCTCAGGACCAG-3' and Smcx_ISH_1r 5'-GACAAGCACCAAAAGCCTTC-3'. The PCR product was cloned using the pGEM-T Easy Vector System I (Promega, Madison, WI, USA) according to the manufacturer's instructions. The vector was linearised and purified prior to sense and antisense *in vitro *transcription in the presence of digoxigenin-UTP (DIG RNA Labeling Kit (SP6/T7), Roche Applied Sciences, Penzberg, Germany).

Whole mount *in situ *hybridisations were carried out according to a previously published protocol [18]. Section *in situ *hybridisation was performed on fresh frozen tissue employing the TECAN GenePaint system as previously described [19].

The experimental procedures that involved mice were approved by the 'Landesamt für Gesundheitsschutz und Technische Sicherheit' (LaGeTSi), Berlin, Germany.

### RNA isolation from patient lymphoblastoid cell lines

Total RNA from lymphoblastoid cell lines was isolated using the Trizol reagent (Invitrogen, Carlsbad, CA, USA). Whole blood was collected and total RNA extracted using the PAXgene Blood RNA System according to the instructions of the manufacturer (PreAnalytiX, Hombrechtikon, Switzerland). The RNA was checked for integrity on ethidium bromide stained agarose gels and the concentration was measured with a NanoDrop ND-1000 Spectrophotometer (NanoDrop Technologies, Wilmington, DE, USA).

### Microarray hybridisation and data analysis

Total RNA (300 ng) from an LCL with a nonsense mutation in KDM5C (Trp1288Ter) and three control cell lines were converted into double stranded, T7 promoter-containing cDNA and transcribed in the presence of biotin using the Illumina TotalPrep RNA Amplification Kit (Ambion, Austin, TX, USA). The labelled cRNA was hybridised to the Sentrix Human-6 Expression Beadchip, which contains probes for >48,000 transcripts (see http://www.illumina.com/ for detailed information). After hybridisation, washing and Cy3-streptavidin (Amersham Biosciences, Piscataway, NJ, USA) staining, the arrays were scanned using an Illumina BeadStation 500 (Illumina, San Diego, CA, USA). The Illumina BeadStudio software was used for analysis and we applied the 'rank invariant' algorithm for normalisation of the expression data.

All micro array data is 'Minimum Information About a Microarray Experiment' (MIAME) compliant and stored under the GEO series access number GSE8252 http://www.ncbi.nlm.nih.gov/geo/. Using the Illumina Sentrix Human-6 v2 platform RNA from 12 patients with mutations in KDM5C and 5 control lymphoblastoid cell lines were analysed.

### cDNA synthesis, QRT-PCR and data evaluation

For use in quantitative PCR 1 μg total RNA was reverse transcribed in the presence of 1.5 microgram random hexamers pdN_6 _(Invitrogen, Carlsbad, CA, USA) and 0.33 mM deoxyribonucleotide triphosphate (dNTP) in a total volume of 30 μl using the Roche (Mannheim, Germany) First Strand cDNA Synthesis Kit. Primers for quantitative RT-PCR were designed by Primer3 software (http://frodo.wi.mit.edu/primer3/; sequences are available upon request). Whenever possible, the amplified cDNA fragments were intron spanning and included the probe sequence from the expression array. PCRs were performed in triplicates of 10 or 30 μl reactions in the presence of SYBR green (Applied Biosystems, Foster City, CA, USA). Quantitations were performed using the absolute quantification setting and a standard curve generated by serial dilution (factor 2) of cDNA.

All calculations for testing differences between groups of patients and controls were performed with the GLM procedure in SAS V.8 (SAS Institute, Cary, NC, USA). Each model included distinct classes of MR patients and the control group as discontinuous independent variable (fixed effect). Since we used one-factorial models no adjustments were performed. Least squares (LS) mean differences were tested by Student t test and the probabilities were calculated for the hypothesis H_0_:LSM(i) = LSM(j).

### WAPDG

WAPDG is an analysis for differentiation between distinct predefined groups by summarising permutated data from weighted expression values of different genes.

Patients and controls were considered as separate groups. For the generation of equally weighted gene expression information, the individual expression values were divided by the total mean of expression values (both groups combined) per gene.

From these data the mean values and SDs per group were used to generate 1,000 permutations per gene for normal distributed expression values within groups based on the 'ran1' and 'gasdev' functions from scientific routines [20] considering only values > 0. This led to a slight positive skew in the distribution of permutation values, which reflected the distribution of the original observations.

For each gene as well as for the combination of all genes the proportion of shared values between groups was determined.

### Northern blot analysis

For Northern blot analyses, poly-A^+ ^RNA was isolated from 75 microgram total RNA by oligo(dT) magnetic bead-based purification (Dynal Biotech, Oslo, Norway). The RNA was separated on a formaldehyde containing gel in 1 × MOPS (Sigma Aldrich, Steinheim, Germany), transferred to a Hybond N+ membrane, and crosslinked by UV light using the autocrosslink settings of a Stratalinker (Stratagene, La Jolla, CA, USA). Probes with an average size between 600 and 1,000 bp were designed to hybridise to the 3' region of the target RefSeq cDNA and PCR amplified from genomic DNA (primer sequences are available upon request). The specificity of the probes was verified by BLAST alignment http://blast.ncbi.nlm.nih.gov/Blast.cgi. The ^32 ^[P]dCTP labelled fragments were purified and hybridised to membranes in UltraHyb buffer (Ambion, Austin, TX, USA) and washed according to the manufacturer's instructions. Subsequently, Northern blots were analysed using a Storm 820 imaging system (APBiotech, Piscataway, NJ, USA). To control for RNA loading, blots were reprobed with a β-actin probe (BioChain, Hayward, CA, USA). Band intensities were quantified with the ImageQuant Version 5.2 (Molecular Dynamics, Sunnyvale, CA, USA), using the β-actin specific band for normalisation.

## Competing interests

The authors declare that they have no competing interests.

## Authors' contributions

LRJ designed the study, performed array hybridisation, PCR analyses, interpreting the data and drafting the manuscript. HB carried out statistical analyses including WAPDG. SR, AT, AN, ARJ, MR, JN, AH, JPF, JC APMB, BH, AN, JG provided patient material and clinical information. RS and SS performed *in situ *hybridisation and interpretation of the data. HHR participated in writing the manuscript and AWK participated in designing the study and drafting the manuscript. All authors read and approved the final manuscript.

## Supplementary Material

Additional file 1 **Table S1**. Influence of mutations in KDM5C on demethylase activity. Click here for file

Additional file 2 **JARID1C mRNA expression in the mouse embryo**. Expression of *JARID1C *as detected in whole mount *in situ *hybridisation at **(a) **Theiler stage (TS)12, **(b) **TS15, **(c) **TS17, and **(d) **TS18. Expression of *JARID1C *is widespread with highest levels in the surface ectoderm, in rostral branchial arches (BA), frontonasal process (FNP), and limb buds (FL, forelimb bud). Higher level of expression is also detected in the headfolds (HF) at TS12 (forebrain anlagen), and from TS17 on, in the somites (So), in the prospective brain (MB, midbrain, Tel, telencephalon), in head ganglia (Tri, trigeminus ganglion) and spinal ganglia (SG). From TS18 on, higher *JARID1C *expression is also observed ventrally in the rostral spinal cord (SC). Midsagittal section *in situ *hybridisation (E) shows highest level of expression in telencephalon (Tel), tooth anlagen (To), heart (Ht), liver (Li), endoderm of gut (G) and umbilical hernia (U), spinal ganglia (SG), ventral horn of the spinal chord (SC) and in cells surrounding the cartilaginous condensations of the vertebral column (VC). Sagittal section hybridisation (F) shows additional expression domains in the metanephros (Me) and olfactory epithelium (OE). The sense probe did not give rise to signals above background levels. Click here for file

Additional file 3 **Northern blot mRNA expression analysis of 4 genes in 12 patients and 5 control cell lines**. The blot was sequentially hybridised with radioactive probes. Using a Storm 820 imaging system (APBiotech, Piscataway, NJ, USA), the expression of four genes was quantified using β-actin expression as normalisation standard. For *CMKOR1*, *MKNK2*, *MYC *and *KDM5B *the mean expression was 3.67-fold, 1.79-fold, 2.54-fold and 2.56-fold higher in the patient group as compared to the control group, respectively. Using the Student t test the level of significance was calculated to be 0.00047, 0.0022, 0.000019 and 0.016, respectively. Click here for file
