# Supplementary material for: A distinctive gene expression fingerprint in mentally retarded male patients reflects disease-causing defects in the histone demethylase KDM5C
Source: Pathogenetics. 2010 Feb 2;3:2. doi: 10.1186/1755-8417-3-2 (PMC2830949; doi:10.1186/1755-8417-3-2)
Supplement: Additional file 1 — Table S1. Influence of mutations in KDM5C on demethylase activity. [file 1755-8417-3-2-S1.DOC]

| Table S1. Influence of mutations in KDM5C on demethylase activity | | | | |
| --- | --- | --- | --- | --- |
|  |  |  |  |  |
| mutation bp | mutation aa | Activity^1^ | |  |
|  |  | H3K4me2 | H3K4me3 | Reference |
| c.202_203insC | R68fsX7 | N.D.^2^ | |  |
| c.260A>G | Asp87Gly | not reduced^3^ | | 12 |
| c.994C>T | Arg332Ter | N.D.^2^ | |  |
| c.1162G>C | Ala388Pro | 14.4 | 45.1 | 11 |
| c.1204G>T | Asp402Tyr | reduced | | 12 |
| c.1924T>C | Phe642Leu | 40.1 | 71.6 | 11 |
| c.2080C>T | Arg694Ter | none | | 12 |
| c.2092G>A | Glu698Lys | reduced | | 12 |
| c.2191C>T | Leu731Phe | 37.2 | 38.1 | 11 |
| c.2248C>T | Arg750Trp | N.D.^4^ | |  |
| c.2252A>G | Tyr751Cys | 51.4 | 56.6 | 11, 12 |
| c.3864G>A | Trp1288Ter | N.D.^2^ | |  |
| 1) Percentual activitivity of H3K4me2 and H3K4me3 demethylase activity, respectively | | | | |
| 2) Truncating mutations are very likely to abolish KDM5C function | | | | |
| 3) KDM5C wih Asp87Gly mutations showed altered cellular localisation | | | | |
| 4) The mutation is located in the conserved C5HC2 zinc-finger domain and is likely to result in reduced demethylase activity | | | | |
