# Supplementary figures and images for: A distinctive gene expression fingerprint in mentally retarded male patients reflects disease-causing defects in the histone demethylase KDM5C
Source: Pathogenetics. 2010 Feb 2;3:2. doi: 10.1186/1755-8417-3-2 (PMC2830949; doi:10.1186/1755-8417-3-2)

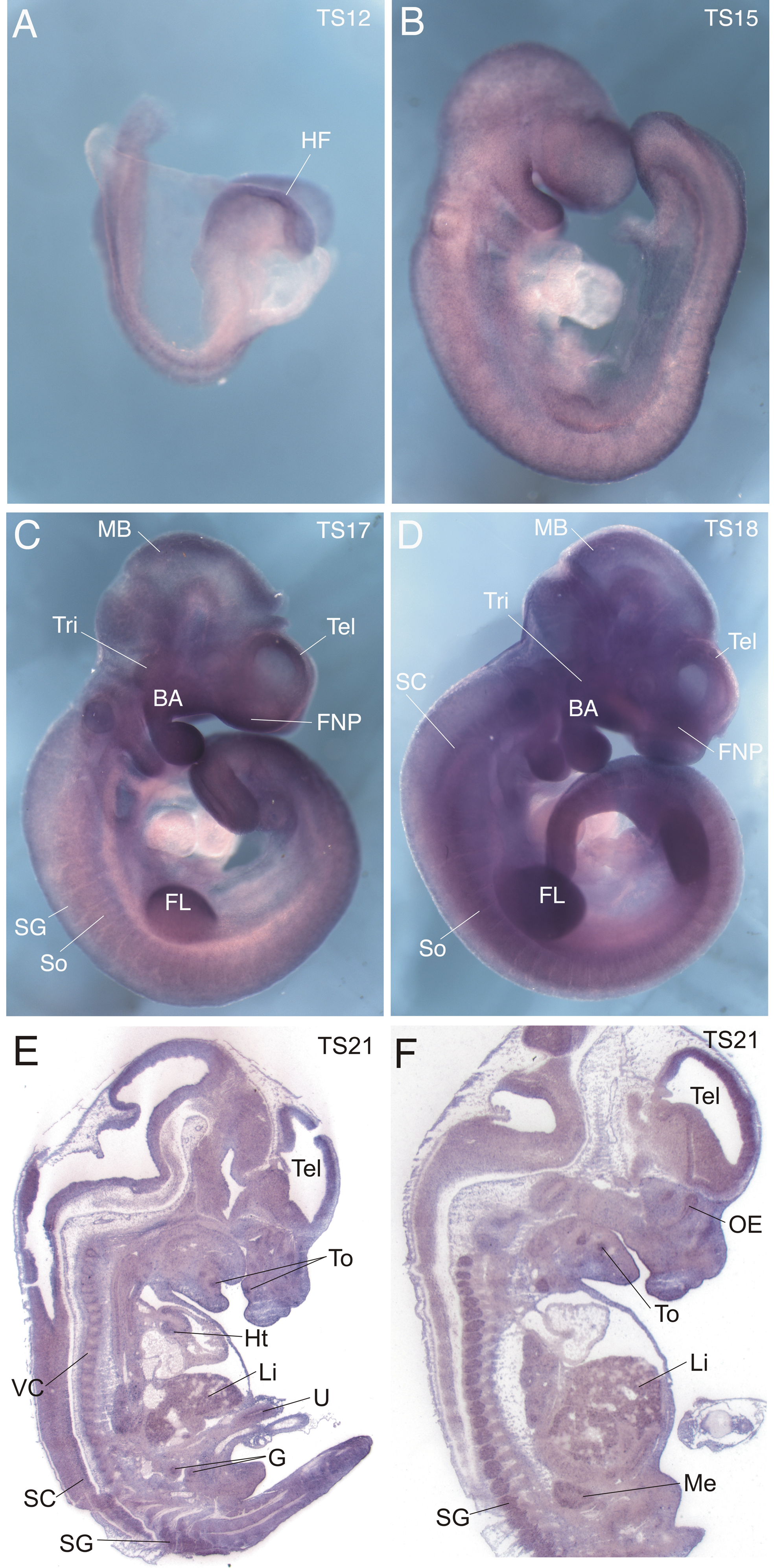

Supplement: Additional file 2 — JARID1C mRNA expression in the mouse embryo. Expression of JARID1C as detected in whole mount in situ hybridisation at (a) Theiler stage (TS)12, (b) TS15, (c) TS17, and (d) TS18. Expression of JARID1C is widespread with highest levels in the surface ectoderm, in rostral branchial arches (BA), frontonasal process (FNP), and limb buds (FL, forelimb bud). Higher level of expression is also detected in the headfolds (HF) at TS12 (forebrain anlagen), and from TS17 on, in the somites (So), in the prospective brain (MB, midbrain, Tel, telencephalon), in head ganglia (Tri, trigeminus ganglion) and spinal ganglia (SG). From TS18 on, higher JARID1C expression is also observed ventrally in the rostral spinal cord (SC). Midsagittal section in situ hybridisation (E) shows highest level of expression in telencephalon (Tel), tooth anlagen (To), heart (Ht), liver (Li), endoderm of gut (G) and umbilical hernia (U), spinal ganglia (SG), ventral horn of the spinal chord (SC) and in cells surrounding the cartilaginous condensations of the vertebral column (VC). Sagittal section hybridisation (F) shows additional expression domains in the metanephros (Me) and olfactory epithelium (OE). The sense probe did not give rise to signals above background levels. [file 1755-8417-3-2-S2.TIFF]

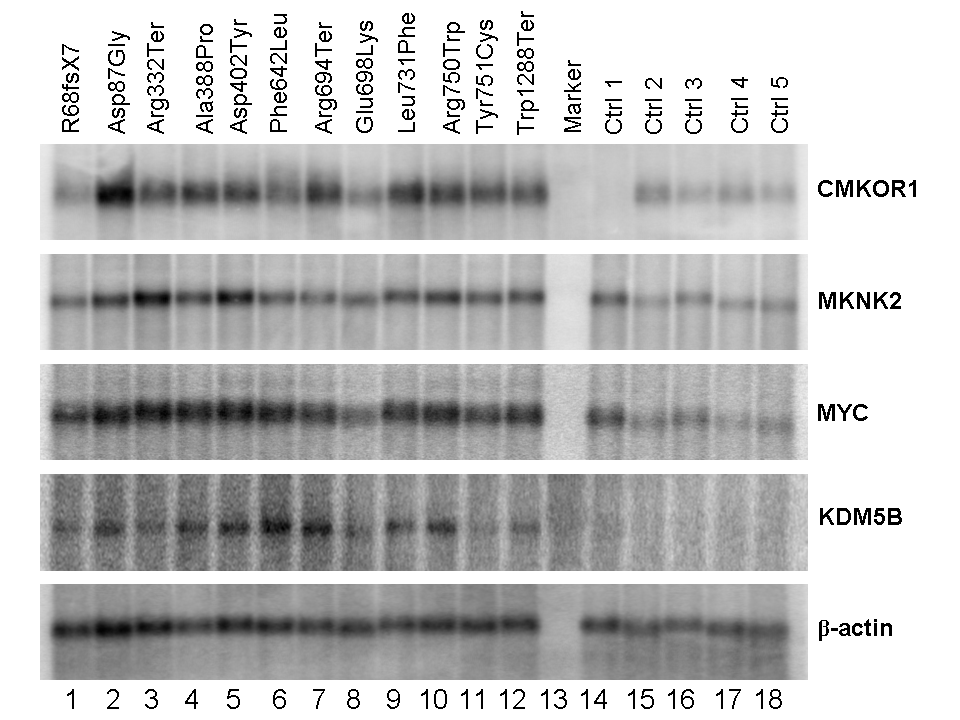

Supplement: Additional file 3 — Northern blot mRNA expression analysis of 4 genes in 12 patients and 5 control cell lines. The blot was sequentially hybridised with radioactive probes. Using a Storm 820 imaging system (APBiotech, Piscataway, NJ, USA), the expression of four genes was quantified using β-actin expression as normalisation standard. For CMKOR1, MKNK2, MYC and KDM5B the mean expression was 3.67-fold, 1.79-fold, 2.54-fold and 2.56-fold higher in the patient group as compared to the control group, respectively. Using the Student t test the level of significance was calculated to be 0.00047, 0.0022, 0.000019 and 0.016, respectively. [file 1755-8417-3-2-S3.TIFF]
